# Consistent RNA expression and RNA modification patterns in cardiotoxicity induced by Matrine and Evodiamine

**DOI:** 10.3389/fphar.2024.1485007

**Published:** 2025-01-09

**Authors:** Guanhua Fang, Yanming Shen, Xinyue Gao, Lele Yang, An Zhu, Dongshan Liao

**Affiliations:** ^1^ Department of Cardiovascular Surgery, Fujian Medical University Union Hospital, Fuzhou, Fujian, China; ^2^ Heart Center of Fujian Medical University, Fuzhou, Fujian, China; ^3^ Key Laboratory of Ministry of Education for Gastrointestinal Cancer, School of Basic Medical Sciences, Fujian Medical University, Fuzhou, China

**Keywords:** m6A (N6-methyladenose), Matrine (M), Evodiamine (evo), cardiotoxicity, RNA editing

## Abstract

Recent research has demonstrated the efficacy of traditional Chinese medicine (TCM) and its active compounds in combating cancer, leading to an increasing utilization of TCM as adjunctive therapy in clinical oncology. However, the optimal dosage of TCM remains unclear, and excessive use may result in cardiotoxicity, which poses a significant health concern for patients undergoing systemic treatment. Therefore, elucidating the underlying mechanisms of cytotoxicity induced by TCM can provide valuable insights for clinical management. In this study, we employed a comprehensive bioinformatics analysis to present sequencing data obtained from AC16 myocardial cells treated with two bioactive derived from botanical drugs: Matrine and Evodiamine. We aim to investigate the dysregulated signaling pathways associated with cardiotoxicity induced by these compounds. Based on our sequencing results, we observed consistent patterns of gene expression and epitranscriptome regulation (m6A and A-to-I modifications) across various drugs-treated AC16 cells when analyzed using KEGG pathway enrichment and gene ontology analyses. Furthermore, m6A writers VIRMA and A-to-I writers ADARB1 is consistent target of Evodiamine and Matrine. In general, our findings suggest that different Chinese botanical drugs induced cardiotoxicity may share common therapeutic strategies.

## Introduction

Although standard anti-cancer medicines can extend a patient’s lifespan, the emergence of drug resistance during disease progression necessitates the administration of additional medications for effective treatment. Traditional medicine has demonstrated its potential as an adjunctive therapy in clinical treatment for anticancer purposes, however, the toxicity and optimal dosage remain uncertain. Among all kind of drug induced cytotoxicity, cardiotoxicity is a fatal, specifically in patients undergoing anticancer treatment. Therefore, we need to understand the mechanism of cytotoxicity induced by traditional medicine and their active compounds, which may provide therapy target to cure patients with cardiotoxicity.

Matrine is a functional compound of the root of *Sophora tonkinensis* Gagnep [Fabaceae] or *Sophora flavescens* Aiton [Fabaceae], which could be used for clinical treatment with a variety of purposes (Zhang et al., 2020), such as alternative medicine for neurological disease (Yin et al., 2017), respiratory diseases (Huang et al., 2014), mental disease and so on. The recent studies have demonstrated that Matrine has been proven to be effective in anticancer therapy as well. For example, the KuShun injunction (active compound Matrine) is widely employed in clinical therapy for various types of cancer (Li et al., 2023), such as breast cancer (Lai et al., 2022), gastric cancer (Huang et al., 2011), hepatocellular carcinoma (Ma et al., 2016), and esophageal cancer (Zhou et al., 2021). Moreover, several experiments have elucidated the underlying mechanisms of Matrine in its anticancer activity. Matrine has been shown to inhibit the cell cycle progression in lung adenocarcinoma cell line A549 and downregulate the expression of VGEGA, thereby reducing metastasis in A549 cells (An et al., 2016). Additionally, Matrine exerts suppressive effects on liver cancer cell Huh‐7 progression and attenuates the Warburg effect by modulating the circROBO1/miR-130a-5p/ROBO1 axis (Huang et al., 2023). Furthermore, Matrine hampers colorectal cancer development in HCT116 and SW480 cells through regulation of the AGRN/Wnt/β-catenin pathway (Huang et al., 2023).

Beside Matrine, a bioactive alkaloid named Evodiamine were used as anticancer medicine in clinical therapy also. Evodiamine is a compound extracted from the fruit of *Tetradium ruticarpum* (A.Juss.) T.G.Hartley [Rutaceae], which is the component of many clinical medicines, such as “Zuo-Jin-Wan (Tang et al., 2016)” and “Wuzhuyu decoction (Wang et al., 2023),” used for treating digestive system diseases and as an adjuvant treatment for cancer (Luo et al., 2021). For instance, the “Wuzhuyu decoction” has been employed clinically for treating chronic non-atrophic gastritis (Hu et al., 2023) and as an adjunctive therapy in gastric cancer (Pan et al., 2009). Evodiamine could slow the growth of cancer by participating the regulation of multiple signaling pathways. For example, Evodiamine could induce the cancer cells apoptosis by caspase-independent and caspase-dependent pathway both. The expression of cyclin A/D could be regulated by Evodiamine to manipulate the cell cycle and inhibit cancer cell proliferation (Hong et al., 2020). The Evodiamine could affect ERK signaling pathway to reduce the metastasis and invasion (Hong et al., 2020).

Although Chinese botanical drugs containing Matrine and Evodiamine are not considered first-line treatments for cancer, they are widely utilized as adjunctive therapies in anticancer regimens. However, the lack of standardized treatment protocols for these drugs as adjunctive therapies can also pose risks to patients due to potential side effects resulting from drug overdosage, the cardiotoxicity induced by the overdosage of Matrine and Evodiamine has been demonstrated in both cell and animal models. In a cell model, treatment of neonatal rat cardiomyocytes with 28.44 μg/mL of Evodiamine for 1 day resulted in reduced cell viability by half compared to the control group (Yang et al., 2017). Similarly, experiments conducted on hiPSC-CMs showed decreased cell viability when cultured with Matrine (Wang et al., 2019), which was attributed to an increase in reactive oxygen species and disruption of calcium homeostasis. Furthermore, treatment of zebrafish with Evodiamine led to paraplasia cordis as animal evidence.

Recent researches have proved that Matrine and Evodiamine cause cardiotoxicity in cell and animal models, but the related signaling pathways are rarely studied. Considering Matrine and Evodiamine are commonly utilized in clinical treatment and the drugs can lead to fatal cardiotoxicity, in this study, we investigated the effects of Matrine and Evodiamine treatment on changes in RNA expression and two epi-transcriptomes (m6A and A-to-I) in myocardial cells AC16. In addition, we found consistent patterns of RNA expression and RNA modification patterns of calls under the cultured with different active compounds, which may provide a therapy target for curing patients with drug-induced cardiotoxicity.

## Methods and materials

### Chemical reagent and cell culture

**FIGURE 1:**
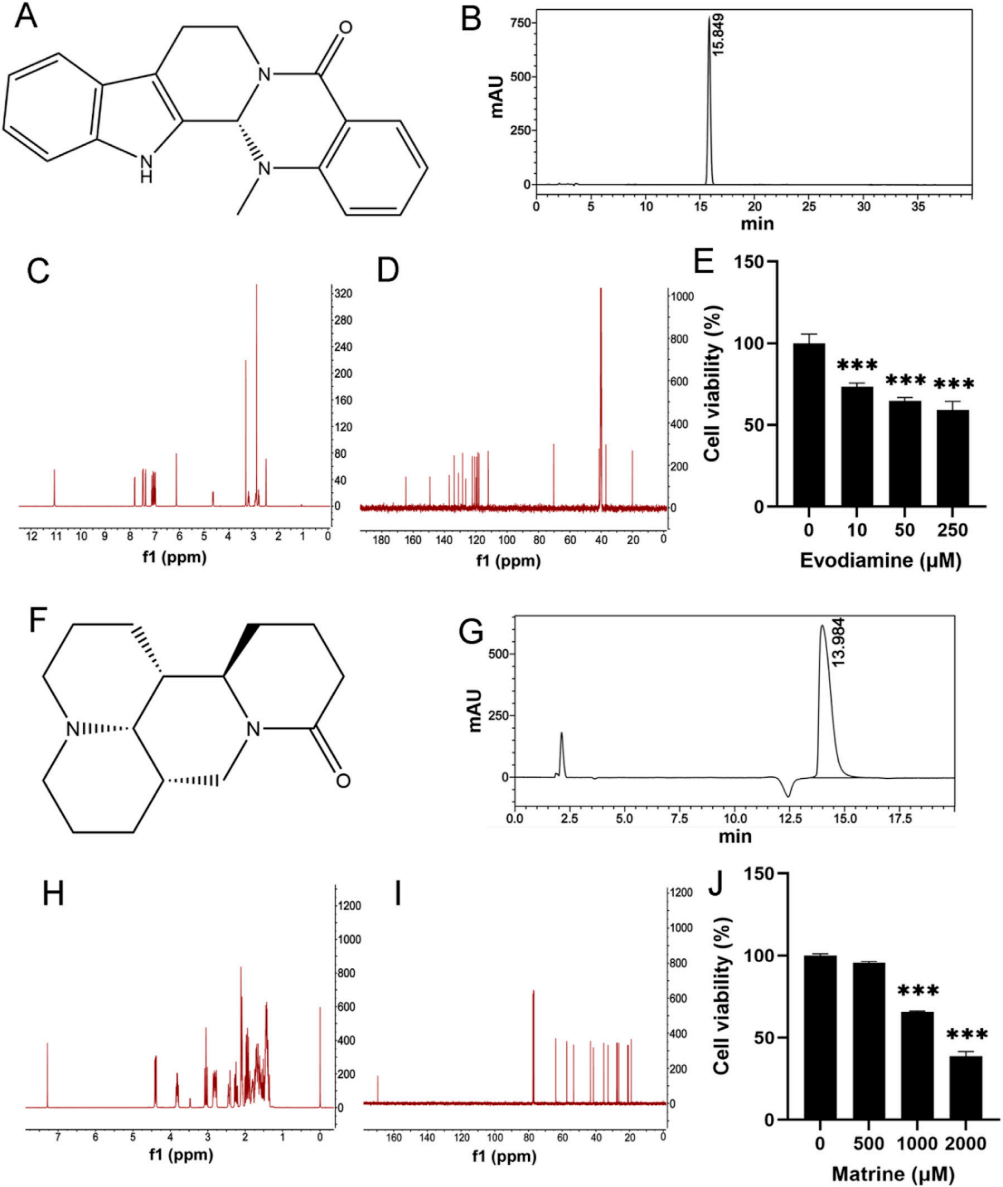
Chemical reagent and Cell viability. **(A, F)** The chemical structures for Evodiamine and Matrine; **(B, G)** The purity of Evodiamine and Matrine; **(C, H)** 400 MHz 1H NMR spectra; **(D, I)** 100 MHz 13C NMR spectra; **(E, J)** Cell viability.

Evodiamine and Matrine were purchased from Chengdu Must Bio-Technology Co., Ltd. (Chengdu, China). The chemical structure of Evodiamine and Matrine (Figures 1A, F) were verified by nuclear magnetic resonance (NMR) spectral analysis (Bruker) (Figures 1C, D, H, I). Based on the detection by high-performance liquid chromatography (Thermo Fisher Scientific Inc.), the purities of Evodiamine and Matrine were 99.80% and 99.99% (Figures 1B, G).

The human AC16 cell line was obtained from the American Type Culture Collection. The cells were cultured in DMEM (BasalMedia, Shanghai, China) supplemented with 10% fetal bovine serum (Gibco, New York, NY, United States), 100 μg/mL streptomycin, and 100 U/mL penicillin G sodium salt at 37°C, humidified atmosphere of 5% CO2. To treat AC16 cells, Evodiamine or Matrine were dissolved in DMSO (Sangon) at concentrations of 10, 50, and 250 μM for 24 h or 500, 1,000, and 2,000 μM for 48 h. The concentration of DMSO in the cell culture was maintained 1%.

### Cell viability

AC16 cells were seeded in 96-well plates with 4,000 cells/well and exposed to 0, 10, 50, and 250 μM Evodiamine for 24 h or 0, 500, 1,000, and 2,000 μM Matrine for 48 h (Figures 1 E, J). Afterward, 10% MTT reagent was added to each well and incubated for 4 h at 37°C. The absorbance was measured at 490 nm using a microplate reader (BioTek, Santa Clara, CA, United States).

### RNA preparation for high throughput sequencing

To maintain a cell viability of 70% in AC16 cells treated with a chemical reagent, we opted for the utilization of 50 μM Evodiamine and 1,000 μM Matrine during cell culture for subsequent high throughput sequencing. HT22 cells were cultured in 6 cm dishes with a cell density of 2 × 10^6^ cells/dish. Subsequently, the cells were subjected to treatment with MA at concentrations of 0 and 50 μM Evodiamine or 1,000 μM Matrine for a duration of 72 h. Following this, TRIzol Reagent (Invitrogen) was utilized for cell lysis. The immunoprecipitation samples contained enrich RNA fragments and input samples were prepared following the protocol from Magna MeRIP m6A kit. All samples were sequenced on a NovaSeq 6,000 platform at Seqhealth Technology Co., Ltd.

### Bioinformatics analysis

The bioinformatics analysis of high-throughput sequencing was conducted using Ubuntu 18.04.6 and R project (version 4.0.3). Details of the parameters used in this study are provided in the Supplementary Material, while the sequencing data can be accessed through Gene Expression Omnibus with accession number GSE274753.

#### RNA-seq

The trim galore was used to remove the low-quality reads from raw data. After that, the Hisat2 (version 2.1.0) (Kim et al., 2019) with default parameters was applied to align the trimmed reads with genome reference (NCBI hg19) to generate sam files, which were convert into bam files by the samtools subsequently (Pertea et al., 2016). The gene expression level was estimated by the stringtie (version 1.3.6) (Pertea et al., 2016) and the differential expression gene was identified by the DESeq2 (Love et al., 2014) (Supplementary Table S1), Genes with a log2FC > 1 or log2FC < −1 and p-value <0.05 were considered to have significant differences. To determine the aberrant gene expression participated signaling pathway (KEGG) of functional regulation (Gene ontology), the significant differential expressed genes were selected and analyzed by the KOBAS (Xie et al., 2011).

#### MeRIP-seq

After the alignment, the enriched m6A modification regions and differential methylated regions were detected by the peak caller exomePeaks2 (Liang et al., 2024) (version 1.9.4), the results were summarized in Supplementary Table S2. The threshold for selecting significant differential methylation regions was set as log2FC > 1 or log2FC < −1 and p-value <0.05. The R package “MateTX” was used to visualize the distribution of modification sites on the message RNA and Long non-coding RNA (Wang et al., 2021). The score of modification sites in the evolutionary conservation was predicted by the ConsRM (Song et al., 2021) web server. The information of diseases associated RNA modification were downloaded from the RMDisease (Song et al., 2023). The significant differential methylated regions were analyzed by the KOBAS also.

#### A-to-I

To detect the RNA editing sites under differential conditions, the RED-ML (Xiong et al., 2017) tools with default parameters were used. The single nucleotide polymorphisms were filtered by dbSNP. The unique A-to-I sites on treated AC16 samples were select for KEGG and Gene ontology analysis by KOBAS.

The interaction of Evodiamine and Matrine with m6A and A-to-I regulators was explored through SYBYL software (Tripos, St Louis, MO, United States). Structure of Evodiamine and Matrine was obtained from PubChem, and crystalline structure of docking proteins was acquired from PDB and UniProt. Then the high precision and semi-flexible docking were performed in SYBYL-X 2.0. The total score encompassing the comprehensive evaluation of hydrophobic complementarity, polar complementarity, solvation terms, and entropic terms. A stable binding between proteins and molecules was considered if the total score surpassed 5. The binding affinity between compounds and proteins were estimated by the AutoDock vina (version 1.1.2) (Trott and Olson, 2010).

### Animal model

**FIGURE 2:**
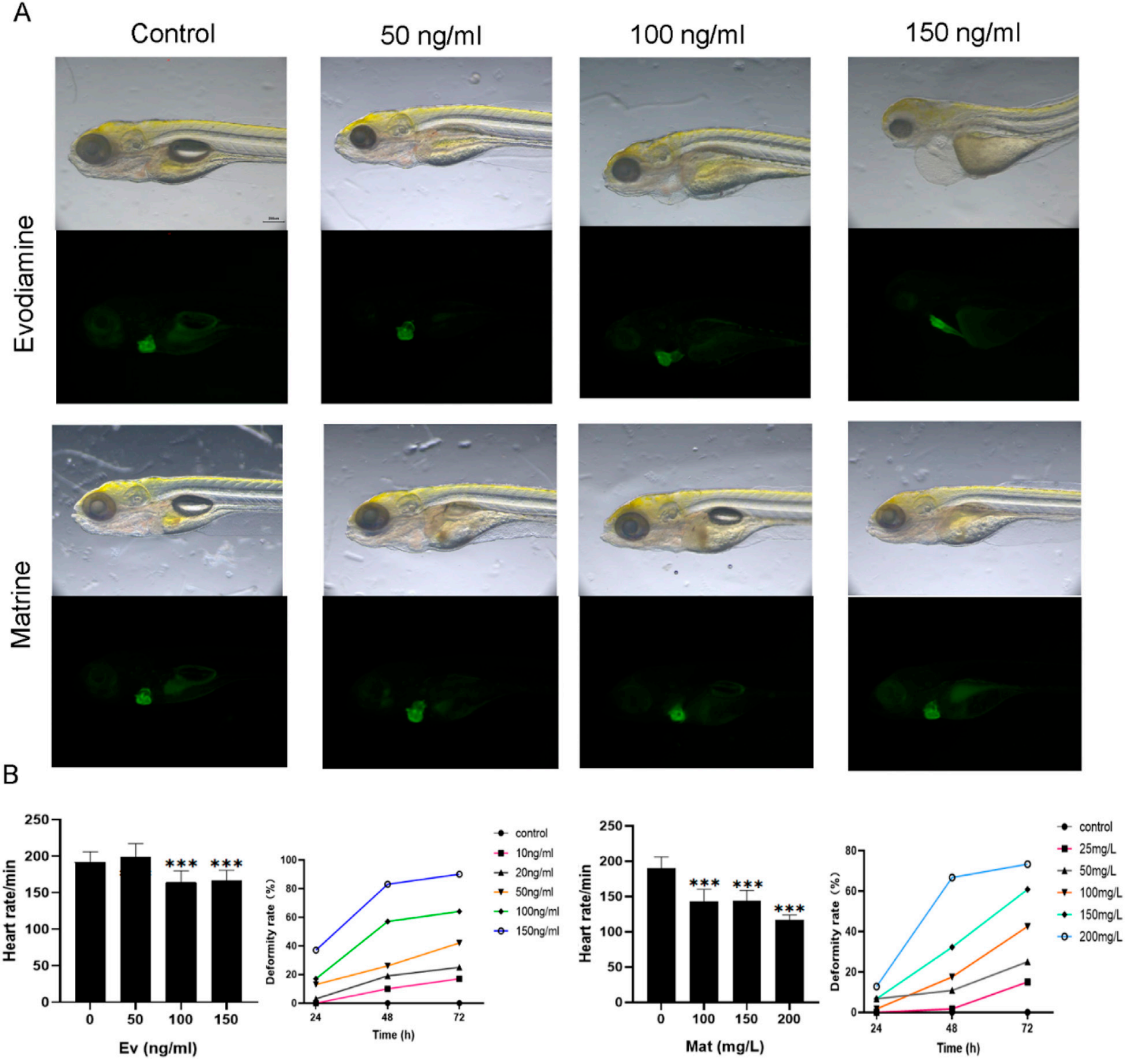
The effect of Matrine and Evodiamine *in vivo*. **(A)** Zebrafish were bred under different conditions with different concentrations of Matrine or Evodiamine, **(B)** The high concentration of Matrine and Evodiamine reduced the heart rate and increased deformity rate of Zebrafish.

The cardiotoxicity induced by Matrine and Evodiamine was validated *in vivo* (Figure 2). The zebrafish are bred under controlled conditions of 28.5°C, with a light-dark cycle of 14 h and a 10-h period of darkness (Figure 2A). The heart rate and deformity rate were counted for zebrafish cultured under different conditions (Figure 2B).

## Result

**FIGURE 3:**
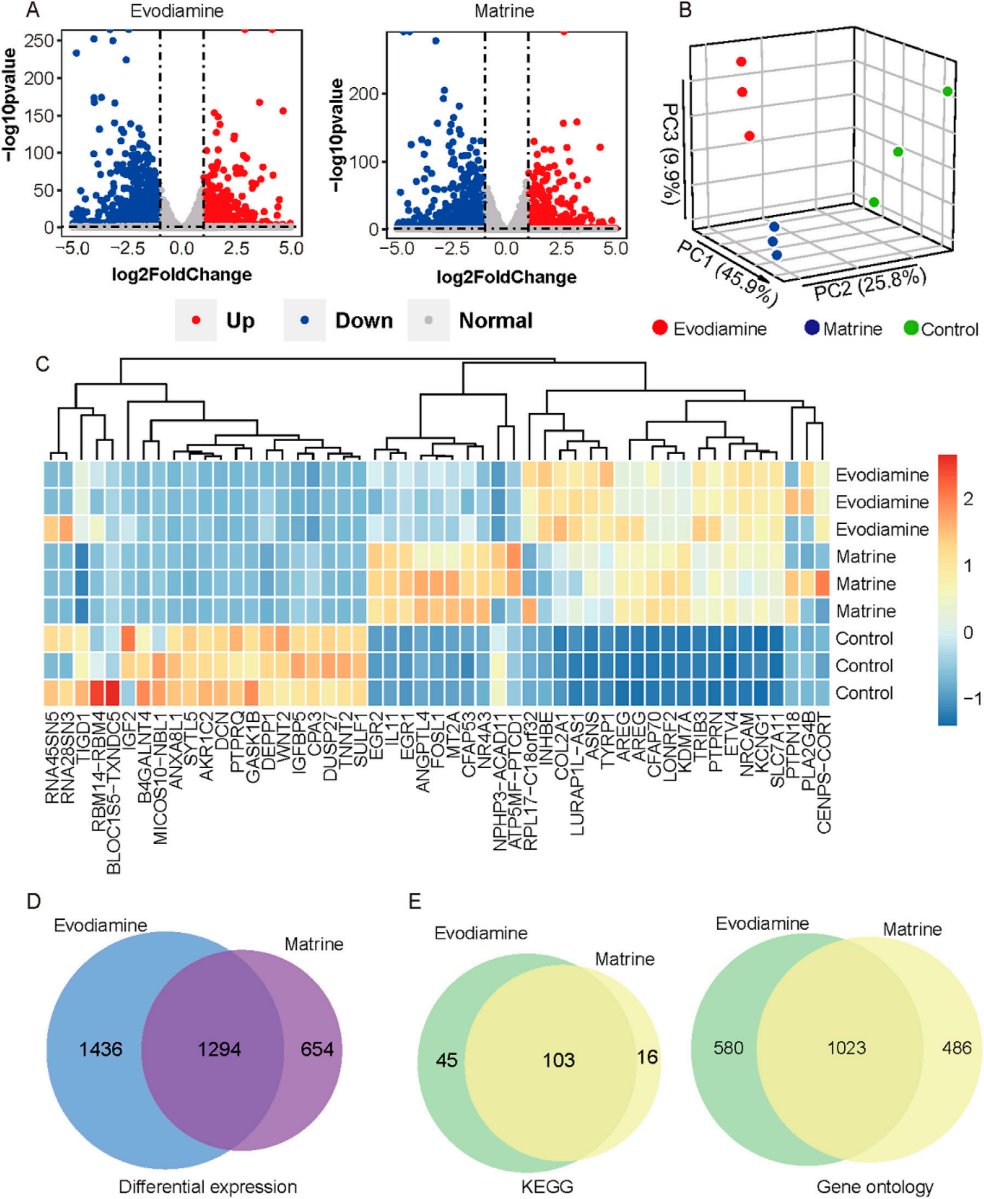
Gene expression profiles on the AC16 and tow cardiotoxic conditions treated Evodiamine and Matrine. **(A)** Volcano plot for differential expression between AC16 and treated Evodiamine and Matrine, respectively **(B)** PCA for three conditions. **(C)** heatmap of top 15 upregulated genes and top 15 downregulated genes in each condition. **(D)** Venn diagram for differential expression. **(E)** Venn diagram to show the similar KEGG and GO enrichment in two cardiotoxic conditions.

Based on the sequencing results, a total of 6,324 and 5,379 differentially expressed genes (log2FC > 1 or log2FC < −1, p-value <0.05) were identified in the Evodiamine and Matrine treated groups (Figure 3A), respectively, when compared to the control samples. The read counts of each sample were utilized for Principal Component Analysis (PCA) to demonstrate the similarity in gene expression status of AC16 cells under different chemical reagents treatment (Figure 3B). Additionally, we present the consistent expression patterns of the top 15 upregulated genes and top 15 downregulated genes induced by Evodiamine and Matrine in AC16 cells, indicating cardiotoxicity (Figure 3C). In total, a set of 1,294 genes exhibited aberrant expression under both drug-induced cardiotoxic conditions (Figure 3D). Furthermore, we observed alterations in 103 signaling pathways and 1,023 gene functions in both drug-induced cardiotoxic conditions (Figure 3E).

**FIGURE 4:**
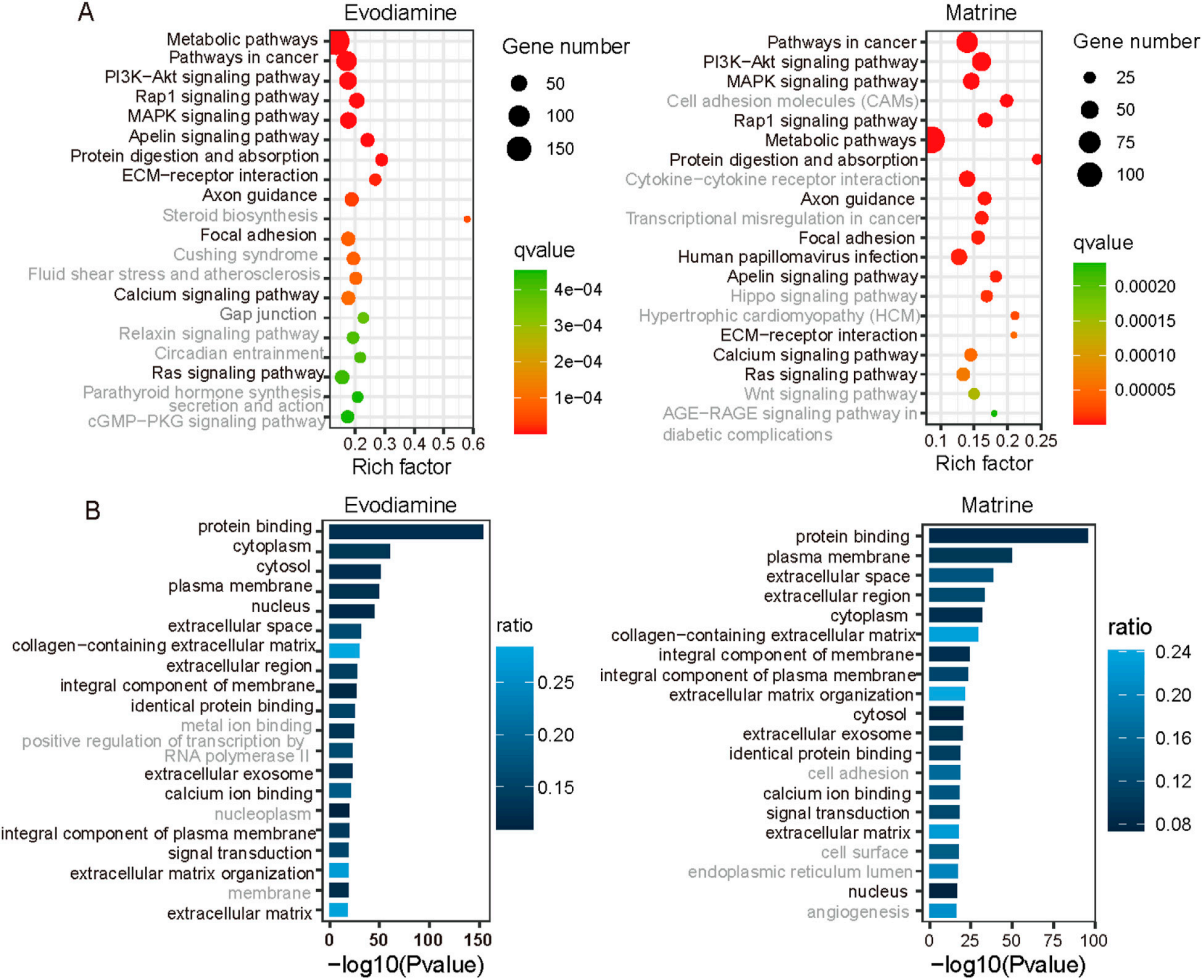
KEGG and gene ontology enrichment based on the differential gene expression profiles. **(A)** KEGG enrichment for Evodiamine and Matrine. **(B)** GO enrichment for Evodiamine and Matrine.

We selected and presented the top 20 significant KEGG pathways and gene ontology results on Evodiamine and Matrine induce cardiotoxicity in Figure 4, respectively. In detail, Evodiamine and Matrine induce cardiotoxicity by the alter identical 12 signaling pathway (Figure 4A), including PI3K-Akt signaling pathway, Rap1 signaling pathway, MAPK signaling pathway, ECM-receptor interaction and calcium signaling pathway which is proven play important roles in the development of cardiac diseases. For example, the activation of MAPK signaling pathway promotes myocardial fibrosis by regulating the generation of AP-1, leading to increased proliferation of fibroblasts and collagen fiber maturation. On the other hands, the inhibition of MAPK signaling could improves heart failure. Among the top 20 gene functions with highest confidence coefficient, 16 gene functions are identical (Figure 4B). These results of KEGG and gene ontology illustrate the cell under the treatment of Evodiamine and Matrine have similar pathways and functions.

**FIGURE 5:**
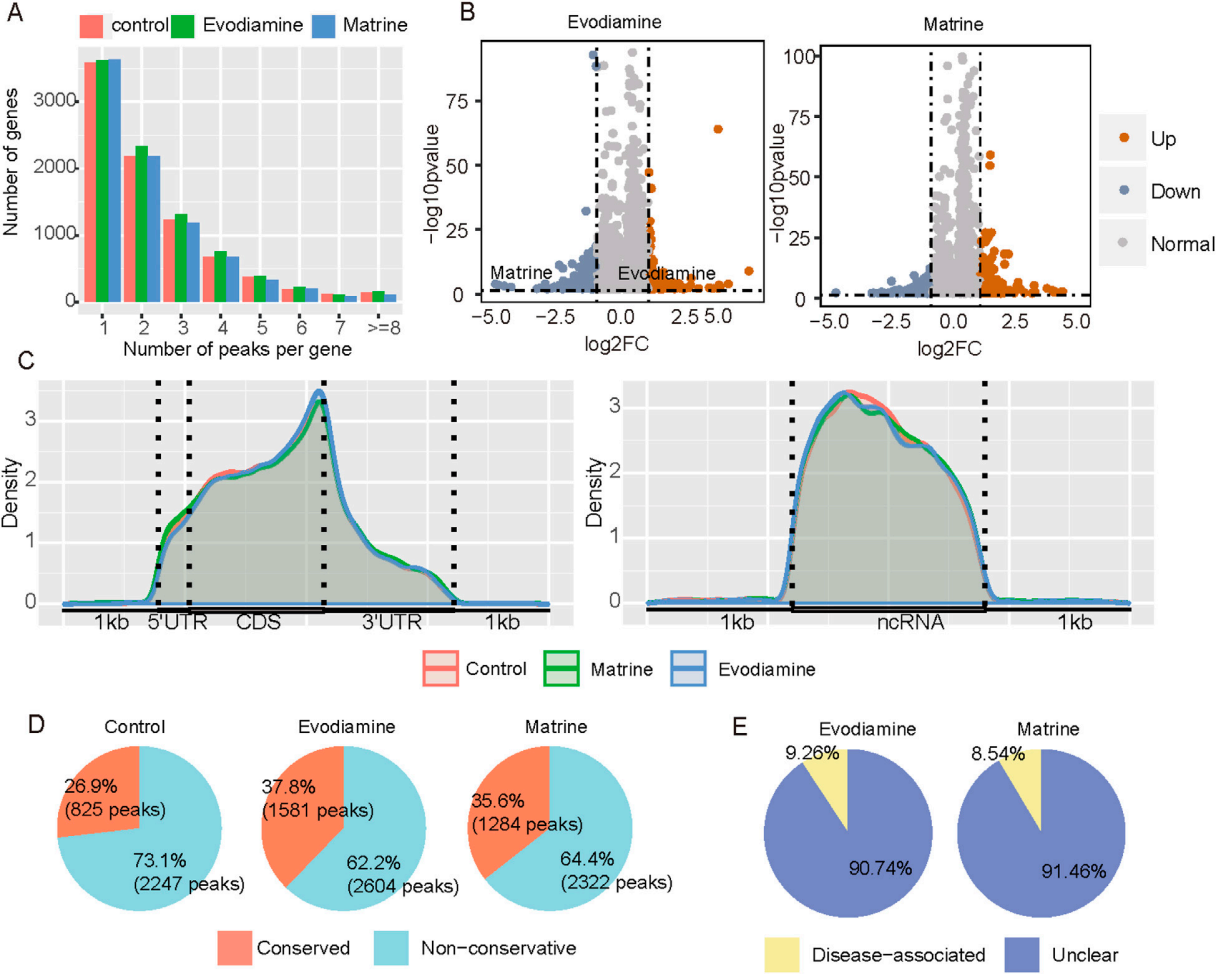
m6A profiles on the AC16 and tow cardiotoxic conditions treated Evodiamine and Matrine. **(A)** The percentage of peak number per gene in different conditions. **(B)** Volcano plot for differential m6A between AC16 and treated Evodiamine and Matrine, respectively. **(C)** The distribution of m6A on mRNA and ncRNA. **(D)** The conservation of m6A sites. **(E)** The disease association with drugs induced differential methylation.

Besides altered gene expression, we also explore the roles of epitranscriptome in the Chinese botanical drugs induced cardiotoxicity. *N*6-methyladenosine is the most prevalent epitranscriptome makers on the human messenger and long non-coding RNA. Compared with the control groups (Figures 5A, B), the increased m6A levels is observed and a total of 464 and 316 differentially methylated peaks (log2FC > 1 or log2FC < −1, p-value <0.05) were identified in the Evodiamine treated with and Matrine treated condition. In regard to the distribution of m6A, the Evodiamine enriched the m6A sites on the end of CDS regions, whereas Matrine raised the m6A sites on the 5′UTR of messenger RNA (Figure 5C). Interesting, the Evodiamine and Matrine suppressed the m6A on the lncRNA both. To explore the potential function of abnormal the m6A sites, the conservation and disease association of m6A were analyzed. After the treatment with the Evodiamine and Matrine, more methyl group was added on the adenosine, which may affect the RNA metabolism to change the signaling pathway and gene functions (Figure 5D). Additionally, 9.26% and 8.54% differential methylated genes contained disease related m6A sites in Evodiamine and Matrine treatment, respectively (Figure 5E).

**FIGURE 6:**
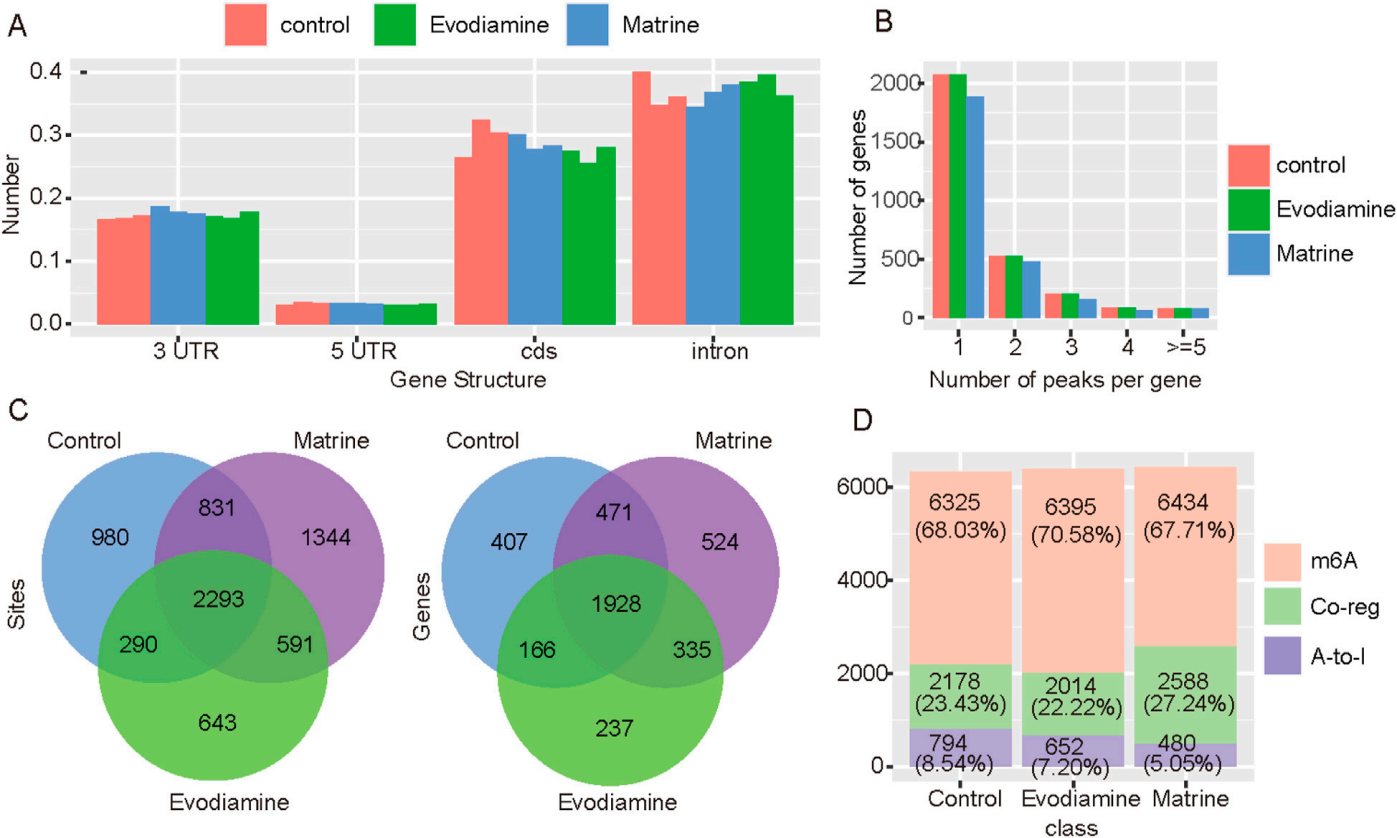
A-to-I profiles on the AC16 and tow cardiotoxic conditions treated Evodiamine and Matrine. **(A)** The change of A-to-I sites on the different gene structures. **(B)** The percentage of A-to-I sites per gene in different conditions. **(C)** The Venn diagram for A-to-I sites and regulated genes in different conditions. **(D)** The percentage of m6A, A-to-I and co-regulated genes.

A-to-I is another type of RNA modification which regulated the gene expression also. We used the RED-ML to estimate the A-to-I status in normal AC16 cell and AC16 with cardiotoxicity. Evodiamine and Matrine suppressed the A-to-I sites on the CDS but enhanced the A-to-I sites on the 3′UTR (Figure 6A). In general, the number of A-to-I sites were reduced in the treatment with Matrine (Figure 6B). Compared with control condition (Figure 6C), samples treated Matrine with have 1,234 new A-to-I sites on the 572 genes, while 1935 new sites on 859 genes were observed in the treatment of Evodiamine. We also investigate the A-to-I and m6A co-regulated genes which take 25% proportion approximately (Figure 6D).

**FIGURE 7:**
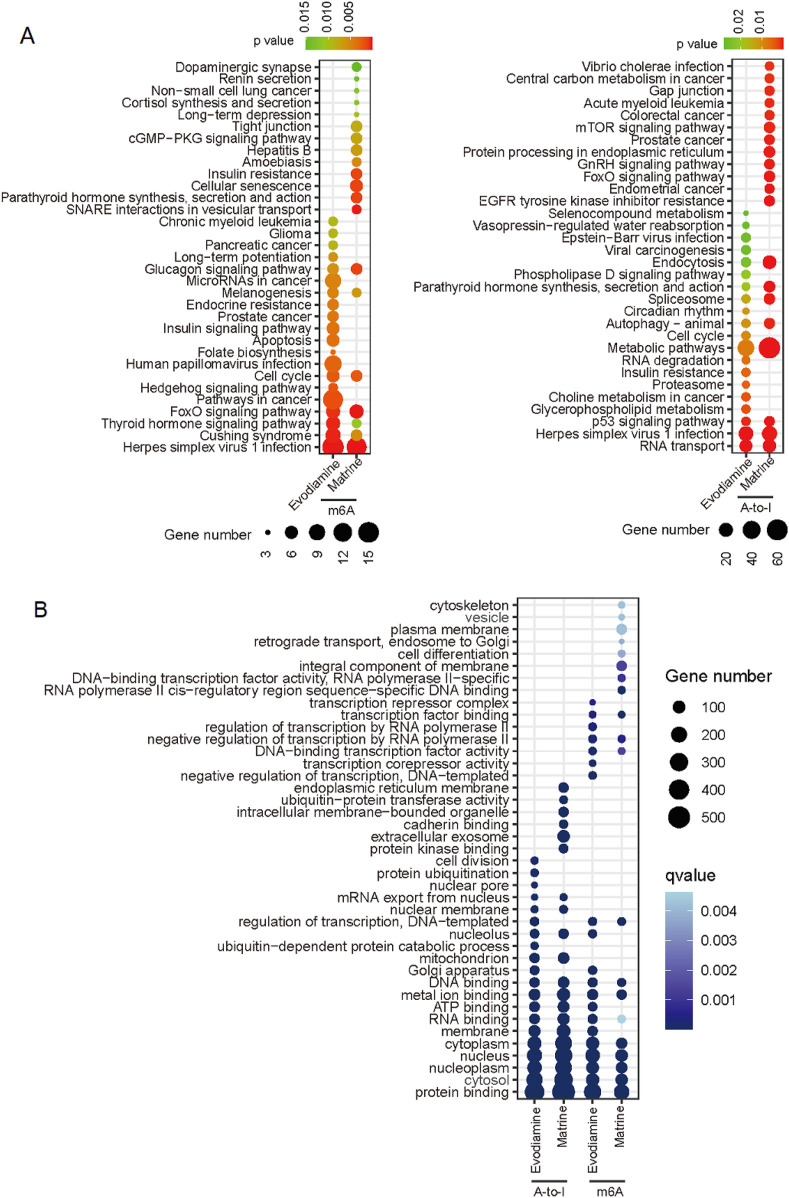
KEGG and gene ontology enrichment based on the differential m6A and A-to-I. **(A)** KEGG enrichment for m6A and A-to-I. **(B)** Gene ontology show the similar function of m6A and A-to-I mediated genes on different drug induced cardiotoxicity.

The RNA modification related signaling pathway and gene function were predicted also. For the m6A modification (Figure 7A), 7 of top 20 significant signaling pathways were consistent in two types of chemical reagents induced cardiotoxicity. For the A-to-I, 8 of top 20 significant signaling pathways were consistent. However, the m6A related pathway is different with the A-to-I regulated pathway, only pathway named ‘Herpes simplex virus 1 infection’ enriched in the A-to-I and m6A regulation both. However, the m6A and A-to-I regulated genes possessed similar gene function based on the gene ontology (Figure 7B).

**FIGURE 8:**
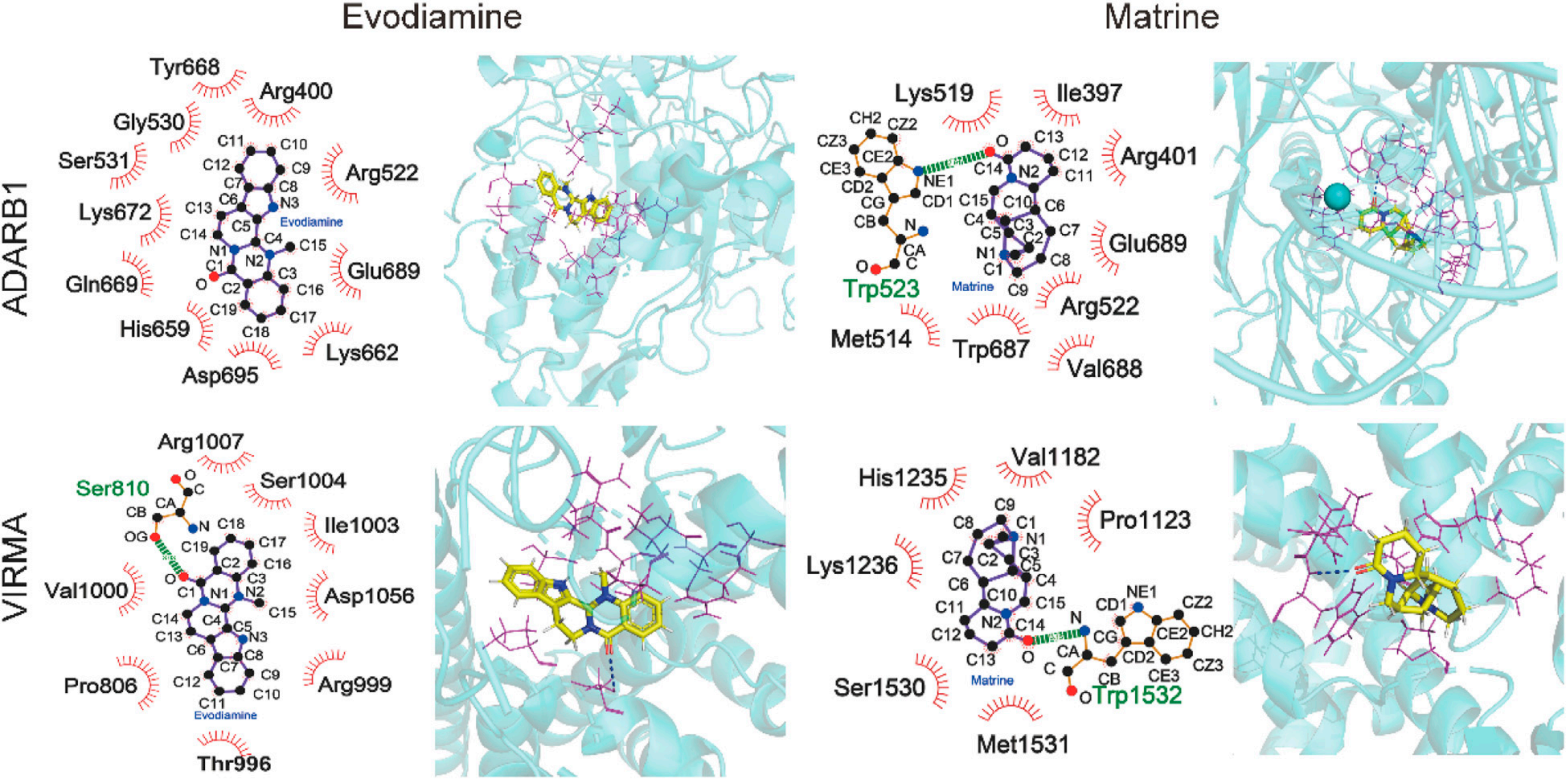
The structure interaction for Evodiamine and Matrine binding with VIRMA and ADARB1.

Considering the Evodiamine and Matrine participated similar m6A or A-to-I regulated signaling pathway and gene functions, we investigate the potential interaction between Evodiamine or Matrine and RNA modification regulators. The potential interaction between drugs and four m6A writers (METTL14, METTL3, WTAP, VIRMA) or three A-to-I writers (ADAR, ADARB1, ADARB2) were estimated by SYBYL software (Table 1). Interestingly, m6A writers VIRMA and A-to-I writers ADARB1 is consistent target of Evodiamine and Matrine, which achieved higher scores (Figure 8). According to the prediction, VIRMA and ADARB1 can form a hydrogen bond with Matrine and exhibit hydrophobic contacts involving 8 and 6 residues, respectively. However, Evodiamine exclusively interacts with VIRMA and ADARB1 through hydrophobic contacts involving 11 and 8 residues, respectively. Furthermore, we employed AutoDock Vina to estimate the binding affinity between compounds and ADARB1 or VIRMA. The lower scores obtained confirm the stable binding of Evodiamine or Matrine with ADARB1 or VIRMA.

**TABLE 1:** The prediction scores for Evodiamine and Matrine interaction with RNA modifications. The total score encompassing the comprehensive evaluation of hydrophobic complementarity, polar complementarity, solvation terms, and entropic terms. Crash was used to assess the structural compatibility between small molecule compounds and protein active pockets. A stable binding between proteins and molecules was considered if the total score surpassed 5. The binding affinity between compounds with ADARB1 or VIRMA were estimated by AutoDock Vina.

	Protein	PDB or Uniprot ID	Total score	Crash	Polar	H-bond number	Residues involved in H-bond formation	Hydrophobic contacts number	Residues involved in hydrophobic contacts	AUTODOCK affinity (kcal/mol)
Evodiamine	ADARB1	5HP2	6.782	−0.227	0.011	0	—	11	Tyr688, Asp695, Gly530, Lys662, Ser531, Glu689, Lys672, Arg522, Gln669, Arg400, His659	−8
	VIRMA	7VF5	5.164	−0.557	0.900	0	—	8	Val1000, Pro806, Thr996, Arg999, Asp1056, Ile1003, Ser1004, Arg1007	−8.3
	METTL14	5IL2	4.841	−0.406	0.001	0	—	7	Ser511, Pro396, Pro397, Asp395, Asn549, Gln550, Tyr406	—
	METTL3	8PW8	4.739	−0.878	1.288	1	Lys513	7	Glu481, Ser511, Ile400, Thr510, Val507, Lys459, Trp457	—
	ADARB2	A4VCH5	4.299	−0.794	1.161	0	—	7	Gly58, Lys70, Pro65, Gln134, Cys69, Ser66, His71	—
	ADAR	7ZJ1	4.142	−0.359	1.395	1	Glu795	7	Asn796, Leu719, Glu718,Ser714, His738, Tyr734, Leu792	—
	WTAP	7YFJ	3.339	−0.356	1.034	1	Tyr64	3	Leu68, Ser71, Leu75	—
Matrine	ADARB1	5HP2	7.046	−0.683	2.252	1	Trp523	8	Lys519, Ile397, Arg401, Glu689, Arg522, Val688, Trp687, Met514	−8
	VIRMA	7VF5	6.093	−0.340	3.099	1	Trp1532	6	His1235, Val1182, Pro1123, Lys1236, Ser1530, Met1531	−6.5
	ADARB2	A4VCH5	5.341	−0.497	2.588	0	—	6	Gln134, Arg59, Lys70, Gly58, Cys69, Pro65	—
	METTL3	8PW8	4.275	−0.368	1.150	0	—	6	Lys459, Glu481, Ser511, His478, Thr510, Thr433	—
	METTL14	5IL2	4.186	−0.989	0.904	0	—	8	Leu409, Tyr406, Pro397, Gly407, Gly548, Gln550, Phe534, Asn549	—
	ADAR	7ZJ1	4.163	−1.648	0.771	0	—	9	Glu733, Glu718, Leu792, Asn796, Glu795, Ser714, Ser737, Tyr734, Leu719	—
	WTAP	7YFJ	2.655	−0.512	0.735	1	Gln72	4	Leu75, Glu74, Ser71, Leu68	—

## Conclusion

In this study, we validated the cardiotoxicity of Matrine and Evodiamine overdosage on zebrafish heart development. We employed RNA-seq and MeRIP-seq techniques to investigate alterations in gene expression and RNA modification patterns induced by Matrine and Evodiamine on AC16 cells. Through comprehensive bioinformatics analysis, we observed consistent changes in gene expression and related signaling pathways under different treatment conditions. Moreover, the m6A and A-to-I profiles of AC16 cells treated with Matrine and Evodiamine exhibited remarkable similarities. Importantly, a higher proportion of evolutionarily conserved adenosines were found to be methylated under cardiotoxic conditions, potentially impacting RNA metabolism and disease induction. Furthermore, we identified consistent KEGG pathway enrichment and gene ontology patterns among the dysregulated m6A- and A-to-I-regulated genes, suggesting that Matrine and Evodiamine may modulate gene expression through shared RNA modification regulators. Finally, molecular docking experiments indicated potential interactions between Matrine/Evodiamine with m6A writer VIRMA protein as well as A-to-I writer ADARB1 protein. These findings underscore the therapeutic potential of targeting RNA modification regulators for mitigating cardiotoxicity induced by Chinese botanical drugs.

## Discussion

Drug-induced heart failure poses a significant health risk, particularly for patients undergoing systemic treatment such as anti-cancer therapy. Emerging research has demonstrated the efficacy of traditional Chinese medicine and its bioactive compounds in anticancer treatment, leading to an increasing utilization of traditional medicine as adjunctive therapy in clinical cancer management. However, the clinical and laboratory research has observed the side effects of overdosage of these Chinese botanical drugs. For instance, Wang and colleagues reported that Matrine activates the Nrf2 antioxidant system to promote ferroptosis in H9c2 cells and induce cardiotoxicity (Wang et al., 2024a). Wang et al. found that Matrine disrupts calcium homeostasis and oxidative stress in hiPSC-CMs (Wang et al., 2019). Evodiamine was reported to affect heart development in zebrafish and reduce cell viability in rat cardiac cells (Yang et al., 2017). These pieces of evidence highlight the potential cardiotoxicity induced by Matrine and Evodiamine; however, there is a lack of omics studies profiling the disruption of gene expression and epitranscriptome caused by these Chinese botanical drugs to further understand their mechanisms for inducing cardiotoxicity. In this study, we present sequencing results obtained from AC16 myocardial cells treated with Matrine and Evodiamine to elucidate the shared aberrant signaling pathways associated with cardiotoxicity induced by Chinese botanical drugs.

RNA-seq and MeRIP-seq is widely used to detect the dynamic gene expression profiles in drug induced cytotoxicity (Zou et al., 2022; Wu et al., 2022; Lin et al., 2024). Based on RNA-seq analysis, we identified genes that were either upregulated or downregulated in AC16 cells cultured with Matrine and Evodiamine. Consistent with the findings of Wang et al., our sequencing results revealed disruptions in the calcium signaling pathway. Furthermore, we observed consistent differential expression of genes and alterations in related signaling pathways or gene functions induced by both compounds. Additionally, we observed consistent patterns of altered RNA modifications caused by Matrine and Evodiamine. Finally, a stable binding between compounds and the m6A writer VIRMA protein or the A-to-I writer ADARB1 was observed in the results of the molecular docking experiment. The results were confirmed by SYBYL and AutoDock Vina. It should be noted that Evodiamine only interacts with VIRMA or ADARB1 through hydrophobic contacts, which is a weak interaction manner. All this evidence suggests that Matrine and Evodiamine may interact with a common mediator to modulate gene expression.

Although this work is the first study to explore the consistent patterns on transcriptome and epitranscriptome of different Chinese botanical drugs induced cardiotoxicity, there are some limitations should be improved in the further study. First, the AC16 cell line was exclusively utilized to investigate the cardiotoxicity induced by Matrine and Evodiamine. Despite being widely employed *in vitro* research, AC16 cells are not an optimal substitute for cardiomyocytes due to their different expression pattern (Onódi et al., 2022). However, it is worth noting that the cardiotoxic effects of Evodiamine and Matrine may not be limited to AC16 cells alone; previous studies have also employed primary rat cardiomyocytes (Yang et al., 2017), H9C2 cell line (Wang et al., 2024a), and human hiPSC-CM (Wang et al., 2019) as *in vitro* evidence. Moreover, we further validated the cardiac toxicity induced by Evodiamine and Matrine using zebrafish as an *in vivo* mode.

To further validate the potential cardiotoxicity induced by Matrine and Evodiamine in human primary cardiac cells or cardiac tissue, we performed gene expression analysis of the CYP450 family in AC16 and a human primary cardiac cell line. The CYP450 enzymes play a pivotal role in drug metabolism. Matrine is metabolized by CYP2A6, CYP2B6, and CYP3A4 (Gong et al., 2015) in hepatocytes, and Evodiamine is metabolized by CYP2C9, CYP1A2, and CYP3A4 (Sun et al., 2013) to protect hepatocytes; these enzymes are expressed at relatively low levels in human primary cardiac cells and AC16 (refer to Supplementary Table S3). This could potentially explain the observed cardiotoxic effects with higher dosage of Matrine and Evodiamine. Furthermore, key regulators associated with cardiotoxicity-related epitranscriptome modifications (m6A writers VIRMA and A-to-I writers ADARB1) exhibit similar gene expression levels in both AC16 cells and human cardiac cells (Table 2), suggesting that our findings may not be specific to AC16 alone. However, it is important to verify all these results using an ideal model such as hiPSC-CM in future studies.

**TABLE 2:** The consistent gene expression level of VIRMA and ADARB1 in human primary cardiac cells and AC16.

baseMean	log2FoldChange	lfcSE	stat	P-value	padj	gene
271.172	0.099	0.286	0.348	0.728	0.810	VIRMA
84.546	−0.551	0.348	−1.580	0.114	0.190	ADARB1

The sequencing results were obtained from Onódi et al. (2022)’s work.

Furthermore, our focus is solely on the acute cardiotoxicity resulting from an overdose of Matrine and Evodiamine. In this context, these drugs modulate multiple signaling pathways that directly impact cardiac cell viability, such as autophagy through the calcium signaling pathway and apoptosis via the PI3K-Akt signaling pathway. However, considering that traditional Chinese medicine is often administered over extended periods, it may also induce long-term cardiotoxicity. The mechanism underlying long-term cardiotoxicity may differ from acute cardiotoxicity since cells or organs can tolerate moderate doses of Evodiamine and Matrine treatment but still affect organ phenotype and function in the long time. For instance, distinct left ventricular systolic and diastolic dimensions were observed with short-term versus long-term doxorubicin injections (O’Connell et al., 2017). Therefore, future studies should investigate potential cardiotoxicity induced by prolonged administration of Matrine and Evodiamine.

Third, the validation based on clinical samples is lacking in this study. Although reports have indicated that the overdose of these Chinese botanical drugs can elevate patients’ heart rate and induce other heart-related symptoms (Qiuhong, 2024), the application of omics studies in clinical research remains limited. Therefore, future investigations should focus on collecting and utilizing clinical samples to validate our findings.

Finally, we only considered the roles of two RNA modifications, m6A and A-to-I, in cardiotoxicity. There are several types of RNA modification are important in the gene regulation also (Ma et al., 2022; Wang et al., 2024b; Zhang et al., 2023), so impact of other types of RNA modifications in cardiotoxicity could be studied also. Additionally, the RNA modification profiles at single nucleotide resolution (Chen et al., 2019; Chen et al., 2021; Zhou et al., 2016) could be considered as a valuable resource for investigating the functional role of RNA modifications in cardiotoxicity induced by Chinese botanical drugs.

## Data Availability

The original contributions presented in the study are publicly available. This data can be found at: NCBI repository, GEO database, accession number GSE274753.
