# Supplementary material for: Consistent RNA expression and RNA modification patterns in cardiotoxicity induced by Matrine and Evodiamine
Source: Front Pharmacol. 2025 Jan 9;15:1485007. doi: 10.3389/fphar.2024.1485007 (PMC11755041; doi:10.3389/fphar.2024.1485007)
Supplement: Supplementary file 2 [file Table3.docx]

Supplement Table 3. The read counts for CYP enzyme in human primary cardiac cells and AC16

|  | human primary cardiac cells | human primary cardiac cells | human primary cardiac cells | AC16 | AC16 | AC16 | gene |
| --- | --- | --- | --- | --- | --- | --- | --- |
| 1 | 0 | 0 | 0 | 0 | 0 | 0 | CYP2C9 |
| 2 | 0 | 0 | 0 | 0 | 0 | 0 | CYP1A2 |
| 3 | 0 | 0 | 0 | 0 | 0 | 0 | CYP2A6 |
| 4 | 0 | 0 | 0 | 0 | 0 | 0 | CYP2B6 |
| 5 | 5 | 0 | 0 | 0 | 0 | 0 | CYP3A4 |

The sequencing results were obtained from Onódi et al. ‘s work[1]

1. Onódi, Z., et al., *Systematic transcriptomic and phenotypic characterization of human and murine cardiac myocyte cell lines and primary cardiomyocytes reveals serious limitations and low resemblances to adult cardiac phenotype.* J Mol Cell Cardiol, 2022. **165**: p. 19-30.
