# Supplementary material for: Consistent RNA expression and RNA modification patterns in cardiotoxicity induced by Matrine and Evodiamine
Source: Front Pharmacol. 2025 Jan 9;15:1485007. doi: 10.3389/fphar.2024.1485007 (PMC11755041; doi:10.3389/fphar.2024.1485007)
Supplement: Supplementary file 4 [file DataSheet1.docx]

#trim galore

trim_galore -j 5 --output_dir output --length 30 --quality 20 --paired seq1.fa seq2.fa

#Hisat2

hisat2 -t -x reference -1 seq1.fa -2 seq2.fa -S output.sam

#samtools

samtools sort -@ 8 -o output.bam input.sam

#stringtie

stringtie -eB -p 20 -G reference.gtf -o output.gtf -A output.txt input.bam

#KOBAS

http://kobas.cbi.pku.edu.cn

#ConsRM

http://180.208.58.19/conservation/

#RMDisease

http://180.208.58.19/RMDisease/

#exomePeak2

exomePeak2(bam_ip = c("IP-con1.bam",

"IP-con2.bam",

"IP-con3.bam"),

bam_input = c("INPUT-con1.bam",

"INPUT-con2.bam",

"INPUT-con3.bam"),

bam_ip_treated = c("IP-T1.bam",

"IP-T2.bam",

"IP-T3.bam"),

bam_input_treated = c("INPUT-T1.bam",

"INPUT-T2.bam",

"INPUT-T3.bam"),

gff = '/hg19.refGene.gtf',

genome =BSgenome.Hsapiens.UCSC.hg19)

#RED-ML

perl red_ML.pl --rnabam INPUT.bam --reference reference.fa --dbsnp dbsnp.vcf --simpleRepeat hg19.simpleRepeat.bed --alu hg19.alu.bed --outdir output
